# Rivaroxaban Suppresses Atherosclerosis by Inhibiting FXa-Induced Macrophage M1 Polarization-Mediated Phenotypic Conversion of Vascular Smooth Muscle Cells

**DOI:** 10.3389/fcvm.2021.739212

**Published:** 2021-11-15

**Authors:** Yanpeng Ma, Yong Zhang, Chuan Qiu, Chunhui He, Ting He, Shuang Shi, Zhongwei Liu

**Affiliations:** ^1^Department of Cardiology, Shaanxi Provincial People's Hospital, Xi'an, China; ^2^Center for Bioinformatics and Genomics, Department of Global Biostatistics and Data Science, School of Public Health and Tropical Medicine, Tulane University, New Orleans, LA, United States; ^3^Department of Cardiology, Fuwai Hospital, National Center for Cardiovascular Diseases, Chinese Academy of Medical Sciences & Peking Union Medical College, Beijing, China

**Keywords:** atherosclerosis, macrophage, vascular smooth muscle cell, polarization, phenotypic conversion, factor Xa, rivaroxaban

## Abstract

**Background:** Factor Xa (FXa) is a mediator initiating and accelerating atherosclerosis (AS). Both macrophage and vascular smooth muscle cells (VSMCs) participate in AS progression. This study was aimed to investigate the mechanisms underlying the effects of the FXa inhibitor rivaroxaban on AS.

**Methods:** Rivaroxaban was administered to AS mice. Primary macrophages were exposed to FXa, treated with rivaroxaban, and transfected with siRNA silencing protease-activated receptor 2 (PAR2), hypoxia-inducible factor 1α (HIF1α), delta-like receptor 4 (Dll4), and Akt. Interaction between macrophages and VSMCs was assessed by co-culturing systems. Atherosclerotic lesions were evaluated by oil red O stain. Fluorescent staining was used to determine the cell phenotypes. Secretions of inflammatory cytokines and collagen were assessed by ELISA and Sircol assays. Western blotting was used to evaluate the protein expression and phosphorylation levels.

**Results:** Rivaroxaban reduced lesion area, accumulation of M1 macrophages, and contractile-synthetic phenotypic conversion of VSMCs in atherosclerotic plaques. FXa exposure induced polarization of macrophages toward M1 and Dll4 high expression, which were inhibited by *PAR2, Akt1*, and *HIF1*α silencing. Rivaroxaban treatment inhibited PAR2/Akt/HIF1α signaling activation and Dll4 expression in FXa-exposed macrophages. By cell-to-cell contact, M1 macrophages induced Notch signaling activation in VSMCs which committed contractile-synthetic conversion. Rivaroxaban treatment and Dll4 silencing incapacitated macrophage in inducing phenotypic conversion of VSMCs upon cell-to-cell contact.

**Conclusion:** Rivaroxaban suppresses AS by inhibiting FXa-induced PAR2/Akt/HIF1α signaling activation-mediated macrophage M1 polarization and high Dll4 expression. These macrophages facilitated VSMCs to perform contractile-synthetic phenotypic conversion upon macrophage-VSMCs cell-to-cell contact.

## Introduction

Vascular smooth muscle cells (VSMCs) are the main cell components of the arterial wall, which are responsible for maintaining the structural and functional integrities of arterial vessels. VSMCs exhibit a range of phenotypes (1). Under normal physiological conditions, VSMCs are quiescent and present contractile phenotypes. When challenged by harmful stimuli, VSMCs lose the contractile properties and get converted into synthetic phenotypes, which is critical in atherosclerotic plaque formation and progression (2). Previous investigations have suggested that the activation of the Notch signaling pathway by its specific ligand Delta-like 4 (Dll4) facilitated VSMCs to accomplish contractile-synthetic phenotypic conversion (3, 4).

Atherosclerosis (AS) is the chronic inflammation of the arterial wall, which is driven by kinds of immune cells. Macrophages are the dominant immune cell type participating in the formation, progression, and evolution of atherosclerotic plaques (5). Macrophages are heterogeneous and characterized by multiple polarization statuses in response to different pathophysiological stimuli. Generally, the classically activated macrophages, namely the M1 phenotype, are believed to contribute to the growth and vulnerability of atherosclerotic plaques due to their pro-inflammatory properties (6).

Mechanisms concerning the regulation of macrophage polarization are complicated. As a G-protein coupled receptor, protease-activated receptor 2 (PAR2) is located on macrophages and takes part in immunity and inflammation *via* multiple signaling pathways, such as NK-κB and p38 MAPK (7). Evidence from previous investigations proved that activation of PAR2 promoted polarization of macrophage M1 (8). A previous investigation pointed out that PAR2 caused activation of the hypoxia-inducible factor 1α (HIF1α) pathway (9). Results from another study proposed that the HIF1α pathway promoted Dll4/Notch signaling (10). Thus, it is reasonable for us to speculate that the activation of the PAR2/HIF1α pathway could lead to the polarization of macrophages M1, which is simultaneously characterized by Dll4 high expression. Our previous investigation suggested that Dll4 on adjacent cells induced Notch signaling activation in VSMCs, which resulted in contractile-synthetic phenotypic conversion (3, 11).

It is now accepted that Factor Xa (FXa) is an activator of PAR2. FXa has been proved to promote the progression of advanced AS (12, 13). Thus, we hypothesized that FXa inhibition can suppress PAR2/HIF1α signaling-induced M1 polarization and Dll4 high expression in macrophages. These macrophages were incompetent of inducing phenotypic conversion of VSMCs *via* activation of the Dll4/Notch pathway. In the current study, FXa inhibitor rivaroxaban was administered to AS mice, and the anti-AS effect was observed. Exogenous FXa exposure was used to induce macrophage M1 polarization. Cell-to-cell contact between macrophages and VSMCs was studied by co-culture systems. In order to further support our hypothesis, direct FXa inhibitor rivaroxaban and specific siRNA against *PAR2, Akt HIF1*α, and *Dll4* were also used to treat the macrophages. We believe that the results from this investigation would increase the knowledge regarding the mechanisms of AS and provide more evidence for the clinical potential application of rivaroxaban in the treatment of AS.

## Materials and Methods

### Mice

C57BL/6J background ApoE^−/−^ mice (Vitalriver, Beijing, China) was used to establish AS in mouse model. Mice were maintained in independent polypropylene cages in a controlled environment with 12 h light-dark cycle, (25 ± 1)°C temperature, and (50 ± 5%) humidity. The mice were free to consume water and food. After being weaned for 2 weeks, the mice were fed with a Western diet (14) (containing 21% milk fat and 0.15% total cholesterol, TD.88137, Harlan Teklad) for 14 weeks to induce AS. Rivaroxaban (Merck) was administered with chow at a dosage of 10 mg/kg body weight per day. The dosage of rivaroxaban was determined based on previous investigations and results from our pilot study (15, 16). After 12 weeks, the mice were sacrificed. The animal study protocols were reviewed and approved by the Institutional Ethics Committee for Animal Use and Experiment of Shaanxi Provincial People's Hospital.

### Plasma Rivaroxaban Concentration Detection

Plasma was acquired after blood samples were processed by centrifugation at 3,000 rpm for 10 min at 4°C. Rivaroxaban concentrations were determined using a high-performance liquid chromatography system by Bayer AG.

### Culture and Treatment of Cells

#### Mouse Peritoneal Macrophage Isolation

Mouse peritoneal macrophages were prepared as stationary phenotype (M0) in accordance with previous descriptions (17). Eight-week-old C57BL/6J mice (SPF class, Animal Experimental Center of Northwestern Polytechnical University) were subjected to 2 ml thioglycollate medium (3%, Solarbio) peritoneal injections. Three days after the injections, the mice were further administered with 3 ml ethylenediaminetetraacetic acid [0.05%, dissolved in phosphate buffered saline (PBS)] and then sacrificed by carbon dioxide (CO_2_) suffocation. The thioglycollate-elicited peritoneal macrophages were then harvested. After centrifugation at 1,200 g for 5 min at 4°C, the cells were collected and further cultured in RPMI-1640 medium (Hyclone) supplemented with 10% fetal bovine serum (FBS, Gibco) for 1 h at 37°C. After the unattached cells were removed by washing with PBS, M0 macrophages were acquired. Macrophages were exposed to FXa (Sigma–Aldrich) at final concentrations of 0, 0.5, 1, and 2 U/L (1 U/L equal to 1.21 nmol/L) for 12 h. Several macrophages were pre-treated with rivaroxaban at a final concentration of 0, 50, 100, and 200 nmol/L, 24 h prior to exposure to FXa treatment at 2 U/L (2.42 nmol/L). Several macrophages were subjected to siRNA transfections 12 h prior to FXa exposure.

#### Isolation of Mouse VSMCs

Mouse VSMCs were isolated according to the protocol from previous investigations (18). After sacrifice, whole aortas were harvested from C57BL/6J mice and further dissected in cold PBS. Fat and connective tissues were removed from the outside, while blood and endothelial cells were scraped off from the inside. The adventitia was removed by incubating the aorta in collagenase II (1 mg/mL, Solarbio) and elastase (1 U/mL, Solarbio) in RPMI-1640 medium for 10 min at 37°C in a humidified atmosphere containing 5% CO_2_ and 95% fresh air. After the adventitia was peeled off, the tissue was further incubated with collagenase II (2.5 mg/mL) and elastase (2.5 U/mL) in RPMI-1640 medium for 2 h at 37°C in the humidified atmosphere, containing 5% CO_2_ and 95% fresh air, till single-cell suspension was reached. Then, the dissociated VSMCs were cultured in RPMI-1640 medium supplemented with 10% FBS, 2 mmol/L glutamine (Sigma-Aldrich), and antibiotics mix (Sigma-Aldrich) at 37°C in a humidified atmosphere containing 5% CO_2_ and 95% fresh air. The VSMCs were subsequently co-cultured with macrophages.

### siRNA Transfections

Expressions of PAR2, HIF1α, and Akt were silenced by RNA interfering (RNAi) technique with siRNA transfections in macrophages. siRNAs were designed and synthesized by TaKaRa, Tokyo, Japan. The targeting sequence of siRNA against *PAR2* was: 5′-AGUCGUGAAUCUUGUUCAATT-3′; for *HIF1*α it was: 5′-CGGCGAAGTAAAGAATCTGAA-3′; for *Akt1* it was: 5′-CCAUGAACGAGUUUGAGUAdTdT-3′; and for *Dll4* it was: 5′-GUGGGUCAGAACUGGUUAUTG-3′. Scrambled siRNA (Thermo, Waltham, MA, USA) was used as a negative control. These siRNAs were transfected into macrophages with HiPerFect siRNA transfection reagent (Qiagen, Duesseldorf, Germany) at final concentrations of 10 mmol/L.

### Cell Co-culture

Both contact and non-contact co-culturing systems were used in this study. Primary VSMCs and treated macrophages were co-cultured with a Millicell-24 Cell Culture Insert Plate system with polyethylene terephthalate (PET) membranes (Millipore, Billerica, MA, USA) according to several previous descriptions (10, 19), which is also demonstrated in **Figure 4A**. For the contact model, macrophages were seeded to the basal surface of the insert at a density of 4 × 10^4^/cm^2^ and incubated overnight for adherence. The insert was then reverted into 6-well-culture plates and incubated in RPMI-1640 medium supplemented with 10% FBS, 2 mmol/L glutamine, and antibiotics mix at 37°C. VSMCs were seeded to the apical surface of the insert density of 4 × 10^4^/cm^2^ and incubated overnight for adherence. The system was then incubated in culture plates in RPMI-1640 medium supplemented with 10% FBS, 2mmol/L glutamine, and antibiotics mix at 37°C for 72 h. For the non-contact model, macrophages were seeded to the bottom of the well-instead of the basal surface of the insert.

### Oil Red O and Immunofluorescence Stains

The hall markers of cell phenotypes of macrophages and VSMCs were indicated by immunofluorescence staining, which was carried out according to our previous descriptions (20). Mice were sacrificed by CO_2_ suffocation. The aortic root was dissected under a microscope and fixed with optimal cutting temperature (OCT, Sakura) embedding medium. Sections of 7-μm were made by cryosectioning. For oil red O stain, the sections were washed with PBS and then processed with an Oil Red O Staining kit (Beyotime, Shanghai, China) as per the protocol provided by the manufacturer. The lesion area was measured. Relative lesion area was calculated as (lesion area/total area) ×100%. Fixed cells or aortic sections were permeabilized with 0.2% Triton X-100 (Sigma–Aldrich). After blocking buffer (Abcam, Cambridge, MA, USA) incubation, the slides were incubated with primary antibodies specific to inducible nitric oxide synthase (iNOS, 1:200, Abcam, Cambridge, MA, USA), F4/80 (1:200, Abcam, Cambridge, MA, USA), myosin heavy polypeptide 11 (MYH11, 1:200, Abcam), and osteopontin (OPN, 1:200, Abcam, Cambridge, MA, USA) at 4°C for 8 h. After PBS washing, the slides were incubated with Alexa Fluor-conjugated secondary antibodies (Abcam). Cell nuclei were stained with 4′, 6-diamidino-2-phenylindole (DAPI, Abcam). An inverted fluorescence microscope (Nikon) was used to observe and capture the images. Image J software was used to analyze the fluorescence images.

### Western Blotting

Cells were rinsed with Dulbecco's PBS on ice and further lysed in a radio-immunoprecipitation assay (RIPA) lysis buffer system (Santa Cruz) supplemented with phenylmethylsulfonyl fluoride (PMSF, Santa Cruz). Total protein was extracted using a Total Protein Extraction Kit (Beyotime). The protein concentration was determined with a BCA kit (Invitrogen, Carlsbad, CA, USA). Protein samples were subjected to 10% sodium dodecyl sulfate polyacrylamide gel electrophoresis (SDS-PAGE) and then transferred to polyvinylidene fluoride (PVDF) membranes. After incubation with blocking buffer (Abcam) for 1 h, the membranes were incubated with primary antibodies specific to PAR2 (1:500, Abcam), Akt (1:500, Abcam), phosphorylated Akt (p-Akt, 1:500, Abcam), 70 kDa ribosomal S6 kinase 1 (p70S6K1, 1:1,000, Abcam), vascular endothelial growth factor (VEGF, 1:1,000, Abcam), HIF1α (1:1,000, Abcam), NICD1 (1:1,000, Abcam), HES1 (1:1,000, Abcam), and GAPDH (1:2,000, Abcam), respectively, at 4°C for 8 h. After washing with tris-buffered saline with Tween 20 (TBST), the membranes were further incubated with secondary antibodies conjugated to horseradish peroxidase (HRP) at room temperature for 1 h. After the membranes were developed with Western Blotting Luminal Reagent (Santa Cruz, CA, USA), the immunoblots were visualized on X-ray films. Image J software was used to analyze the optic densities of the blots.

### Enzyme-Linked Immunosorbent Assay

Pro-inflammatory cytokines including interleukin (IL) 1β, IL6, and tumor necrosis factor-alpha (TNF-α) in cell culture medium were determined by ELISA. Commercial mouse IL1β ELISA kit (Solarbio, Beijing, China), mouse IL6 ELISA kit (Solarbio), and mouse TNFα ELISA kit (Beyotime) were used to accomplish the measurements according to the protocol provided by the manufacturers.

### Sircol Collagen Assay

Sircol collagen assay (SCA) was used to detect the secretion of collagen in the cell medium of VSMCs. This assay was performed by using an SCA kit (Biocolor Life Science, Carrickfergus, UK) as per the instructions provided by the manufacturer.

### Statistics

Data acquired in this study were presented in a (mean ± SD) manner and analyzed by using SPSS software (Ver 17.0). The normality of data was examined by Shapiro–Wilk test. Student's *t*-test and one-way ANOVA were used to analyze the difference between two and multiple groups if the data were normally distributed. Mann–Whitney *U*-test was employed if the data was not normally distributed. When *P* < 0.05, the compared differences were considered statistically significant.

## Results

### Rivaroxaban Administration Reduced Plaque Area, M1 Macrophage Infiltration, and VSMCs Phenotypic Conversion in AS Lesions

**Figure 1:**
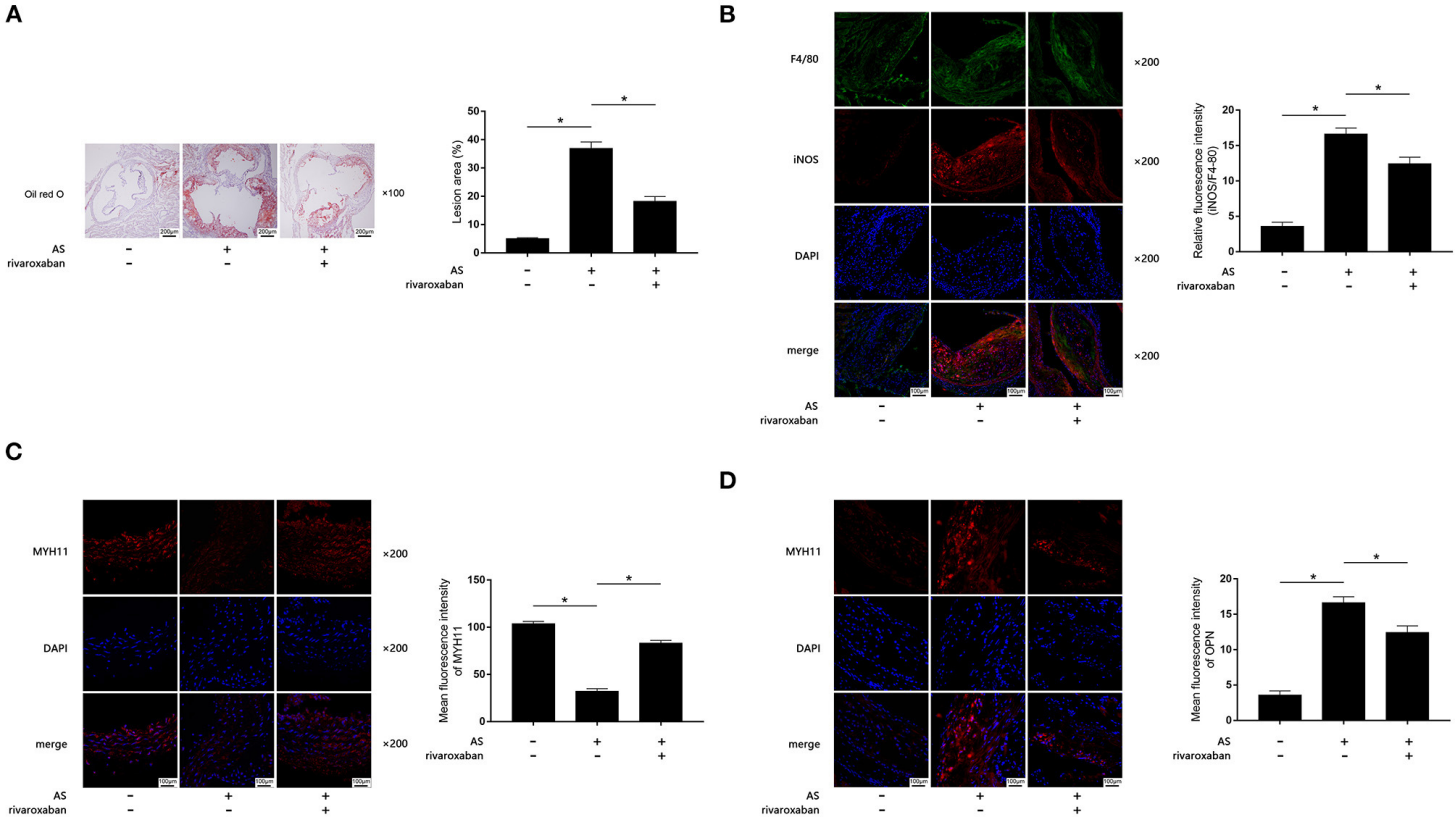
**(A)** Atherosclerotic plaques are shown by oil red O stain of aorta root of ApoE^−/−^ mice, which are demonstrated on the left panel. Columns on the right side indicate relative plaque area in control, atherosclerosis (AS) mice, and AS mice treated with rivaroxaban. **(B)** Immunofluorescence stains of F4/80, iNOS, DAPI, and their merged images are demonstrated. Columns indicate the relative fluorescence intensity of iNOS (normalized to F4/80) in aorta root harvested from control, AS mice, and AS mice treated with rivaroxaban. **(C)** Immunofluorescence stains of MYH11, DAPI, and their merged images are demonstrated. Columns indicate the mean fluorescence intensity of MYH11 in aorta root harvested from control, AS mice, and AS mice treated with rivaroxaban. **(D)** Immunofluorescence stains of OPN, DAPI, and their merged images are demonstrated. Columns indicate the mean fluorescence intensity of OPN in aorta root harvested from control, AS mice, and AS mice treated with rivaroxaban (data were presented as mean ± SD, *n* = 6, Mann–Whitney *U*-test was used, *P < 0.05).

The body weight, plasma rivaroxaban concentration, and lipid profile of mice when they were sacrificed are demonstrated in Table 1. As demonstrated in Figures 1A–D, when compared with the control, obviously increased atherosclerotic plaque lesion area, relative iNOS expression (normalized to F4/80), and decreased MYH11 expression were found in AS lesions of the aortic root of ApoE^−/−^ mice. iNOS is generally considered as the hall marker of M1 polarized macrophages. MYH11 and OPN are recognized as hall markers of the contractile and synthetic phenotypes of VSMCs. Administration of rivaroxaban, however, significantly decreased plaque area, iNOS, and OPN expression, but increased MYH11 expression in AS lesions of the aortic root of ApoE^−/−^ mice. These results suggested that rivaroxaban inhibited plaque progression, possibly by manipulating macrophage M1 polarization and phenotypic conversion of VSMCs.

**Table 1:** Bodyweight, plasma rivaroxaban concentration, and lipid profiles.

**Treatment**	**WTD**	**–**	**+**	**+**
	**Riva**	**–**	**–**	**+**
Bodyweight (g)		30.19 ± 0.41	39.12 ± 0.60	38.69 ± 0.61
plasma riva (mmol/L)		<2.0	<2.0	30.31 ± 1.90
TC (mmol/L)		3.30 ± 1.02	18.86 ± 1.60	19.27 ± 1.98
LDL (mmol/L)		1.65 ± 0.61	8.83 ± 0.80	9.26 ± 0.63
TG (mmol/L)		0.32 ± 0.04	0.70 ± 0.05	0.71 ± 0.04

*WTD, western-type diet; Riva, rivaroxaban; TC, total cholesterol; LDL, low-density lipoprotein; TG, triglycerides*.

### Factor Xa Leads to Macrophage Polarization Toward M1 by Activating PAR2

**Figure 2:**
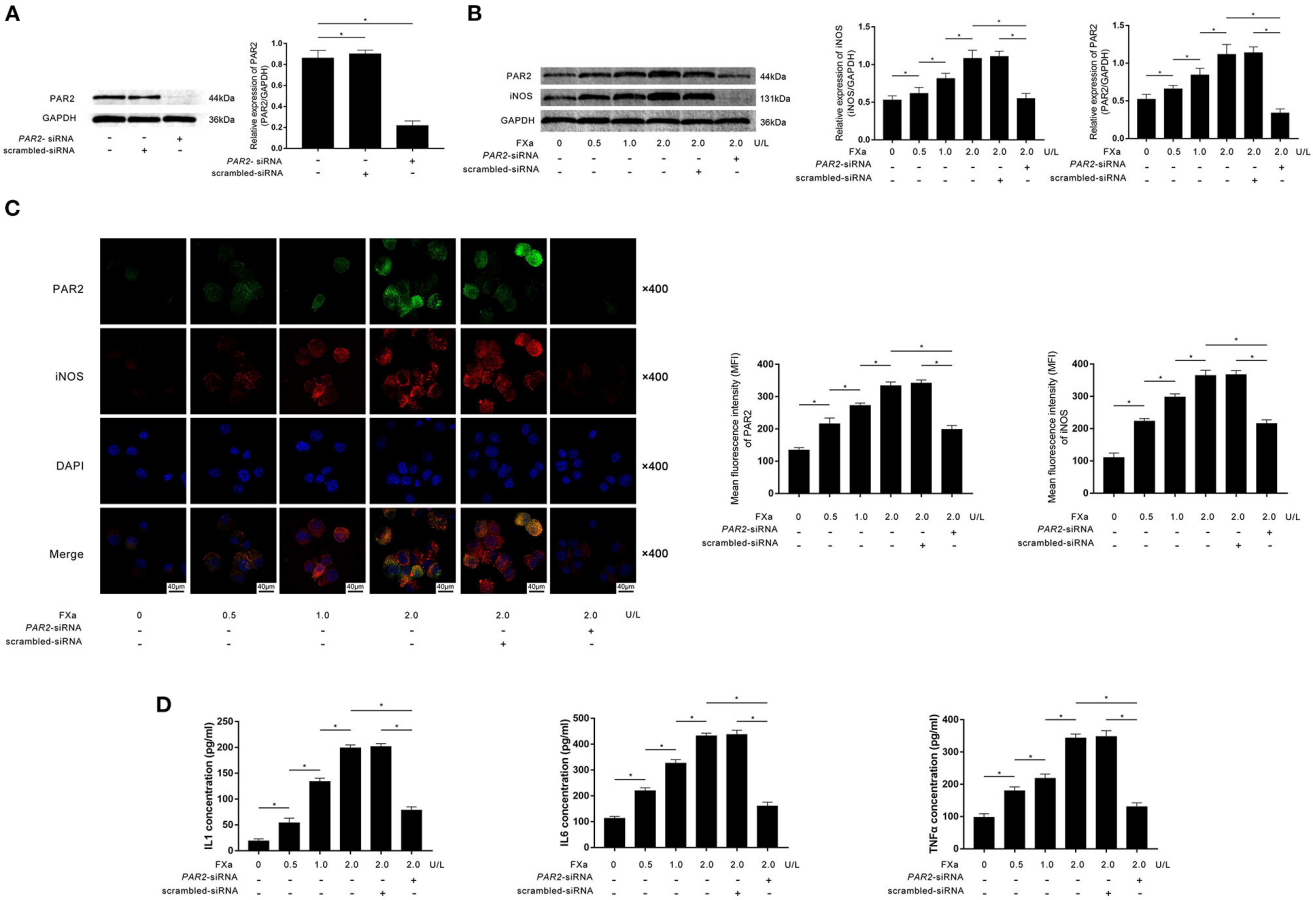
**(A)** Immunoblots of PAR2 and GAPDH are demonstrated. Columns indicate relative expression levels of PAR2 in macrophages transfected with *PAR2*-siRNA or scrambled siRNA. **(B)** Immunoblots of PAR2, iNOS, and GAPDH are demonstrated. Columns indicate the relative expression levels of iNOS and PAR2 in macrophages exposed to FXa at 0, 0.5, 1.0, and 2.0 U/L. Macrophages treated with FXa at 2.0 U/L were also transfected with *PAR2*-siRNA and scrambled siRNA. **(C)** Immunofluorescence stains of PAR2, iNOS, DAPI, and their merged images are demonstrated. Columns indicate the MFI of PAR2 and iNOS in macrophages exposed to FXa at 0, 0.5, 1.0, and 2.0 U/L. Macrophages treated with FXa at 2.0 U/L were also transfected with *PAR2*-siRNA and scrambled siRNA. **(D)** Columns indicate the concentrations of IL1, IL6, and TNFα in the cell culturing medium of macrophages exposed to FXa at 0, 0.5, 1.0, and 2.0 U/L. Macrophages treated with FXa at 2.0 U/L were also transfected with *PAR2*-siRNA and scrambled siRNA (data were presented as mean ± SD, *n* = 6, Student's *t*-test and ANOVA, *P < 0.05).

Specific siRNA against *PAR2* was transfected into M0 macrophages. As demonstrated in Figure 2A, the transfection significantly reduced the expression of PAR2. M0 macrophages were then exposed to exogenous FXa at 0, 0.5, 1, and 2 U/L for 12 h. As demonstrated in Figures 2B,C, FXa stimulation significantly increased the expression levels of PAR2 as well as iNOS, which is believed to be the hall marker of M1 phenotype in a concentration-dependent manner. The exposure also increased the secretion of inflammatory cytokines including IL1β, IL6, and TNFα in an FXa concentration-dependent manner (Figure 2D). As shown in Figure 2C, the transfection of *PAR2*-siRNA significantly decreased iNOS expression in macrophages exposed to FXa at 2 U/L. The transfection also significantly reduced the production of IL1β, IL6, and TNFα from macrophages (Figure 2D).

### PAR2/Akt/HIF1α Pathway Activation Increased Dll4 Expression in FXa-Exposed Macrophages

**Figure 3:**
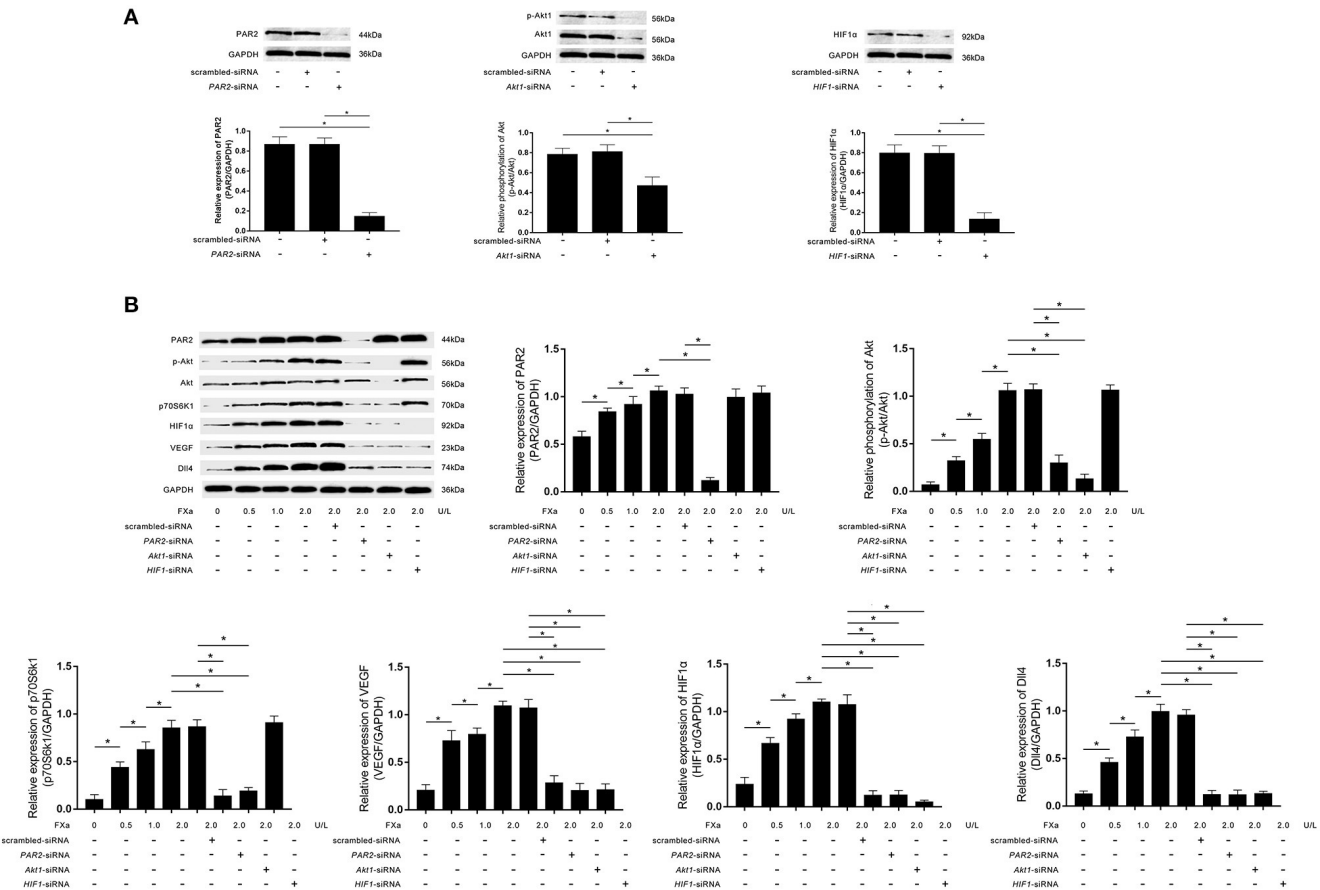
**(A)** Immunoblots of PAR2, phosphorylated Akt1 (p-Akt), Akt1, HIF1α, and GAPDH. Columns indicate the relative expression levels of PAR2 and HIF1α as well as the relative phosphorylation level of Akt in macrophages transfected with *PAR2*-siRNA, *HIF1*α-siRNA, and *Akt1*-siRNA, respectively. **(B)** Immunoblots of PAR2, p-Akt, Akt, p70S6K1, HIF1α, VEGF, Dll4, and GAPDH. Columns indicate the relative expression levels of PAR2, p70S6K1, HIF1α, VEGF, and Dll4, as well as the relative phosphorylation level of Akt in macrophages exposed to FXa at 0, 0.5, 1.0, and 2.0 U/L. Macrophages treated with FXa at 2.0 U/L were also transfected with *PAR2*-siRNA, *Akt1*-siRNA, *HIF1*-siRNA, and scrambled siRNA (data were presented as mean ± SD, *n* = 6, Student's *t*-test and ANOVA, *P < 0.05).

As demonstrated in Figure 3A, *PAR2*-siRNA, *Akt1*-siRNA, and *HIF1*α-siRNA effectively inhibited the phosphorylation of Akt and expressions of PAR2 and HIF1α in macrophages. FXa exposure significantly increased the expression levels of PAR2, p70S6K1, HIF1α, VEGF, Dll4, as well as the phosphorylation levels of Akt in macrophages in a concentration-dependent manner (Figure 3B). As shown in Figure 3B, *PAR2*-siRNA transfection reduced the phosphorylation of Akt and the expression levels of p70S6K1, HIF1α, VEGF, and Dll4. *Akt1*-siRNA transfection reduced the expression levels of p70S6K1, HIF1α, VEGF, and Dll4. *HIF1*α-siRNA transfection reduced the expression levels of VEGF and Dll4 in macrophages exposed to FXa at 2 U/L.

### FXa-Exposed Macrophage Induced Contractile-Synthetic Phenotypic Conversion of VSMCs in Contact Co-culturing Model by Activating Notch Pathway in VSMCs

**Figure 4:**
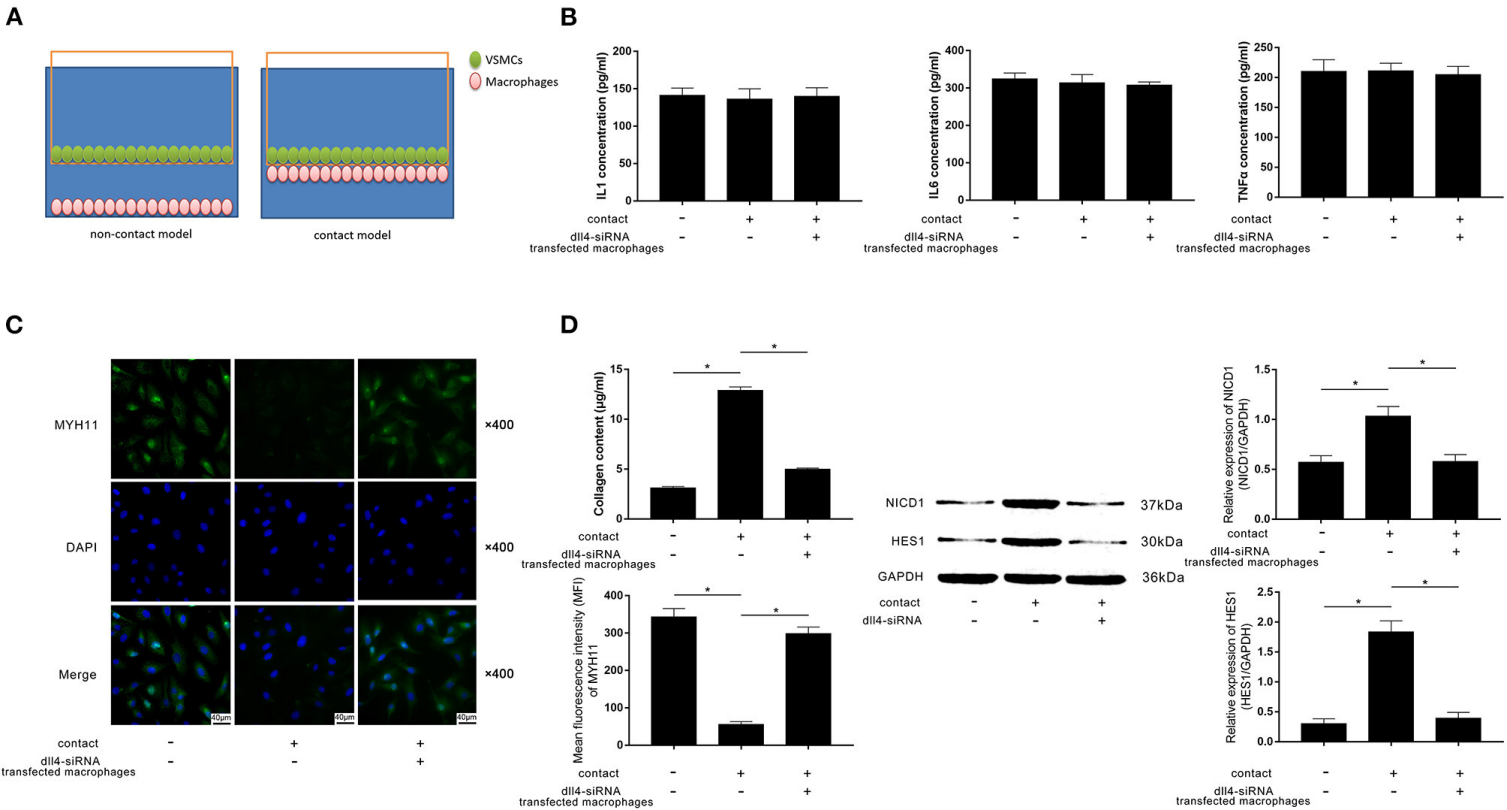
**(A)** Schematic diagram of the non-contact and contact co-culturing models of macrophages and VSMCs is demonstrated. VSMCs in the contact co-culturing model were co-cultured with FXa- exposed macrophage transfected with or without *Dll4*-siRNA. **(B)** Columns indicate the detected concentrations of inflammatory cytokines including IL1, IL6, and TNFα in the non-contact and contact co-culturing model systems. **(C)** Immunofluorescence stains of MYH11 and DAPI and their merged images of VSMCs. Columns indicate the MFI of MYH11 and collagen content of VSMCs from the non-contact and contact co-culturing models. **(D)** Immunoblots of NICD1, HES1, and GAPDH. Columns indicate the relative expression levels of NICD1 and HES1 in VSMCs from the non-contact and contact co-culturing models (data were presented as mean ± SD, *n* = 6, Student's *t*-test and ANOVA, *P < 0.05).

A contact co-culture model was used to study the cell-to-cell effects between FXa-exposed macrophages and VSMCs. A non-contact co-culture model was used as a control. Diagrams of the models are demonstrated in Figure 4A. As evidenced by ELISA, differences in concentrations of IL1β, IL6, and TNFα in medium from contact and non-contact co-culture models were not statistically significant (Figure 4B). Compared with the non-contact model and Dll4-silenced macrophages in the contact model, evidenced by reduced MYH11 expression and collagen content, VSMCs in the contact model committed contractile-synthetic phenotypic conversion (Figures 4C,D). The expression levels of NICD1 and HES1, which are considered as the markers of Notch pathway activation, were upregulated in VSMCs contacted with macrophages not transfected with *Dll4*-siRNA (Figure 4D).

### Rivaroxaban Suppressed FXa-Induced Macrophage M1 Polarization by Inhibiting PAR2

**Figure 5:**
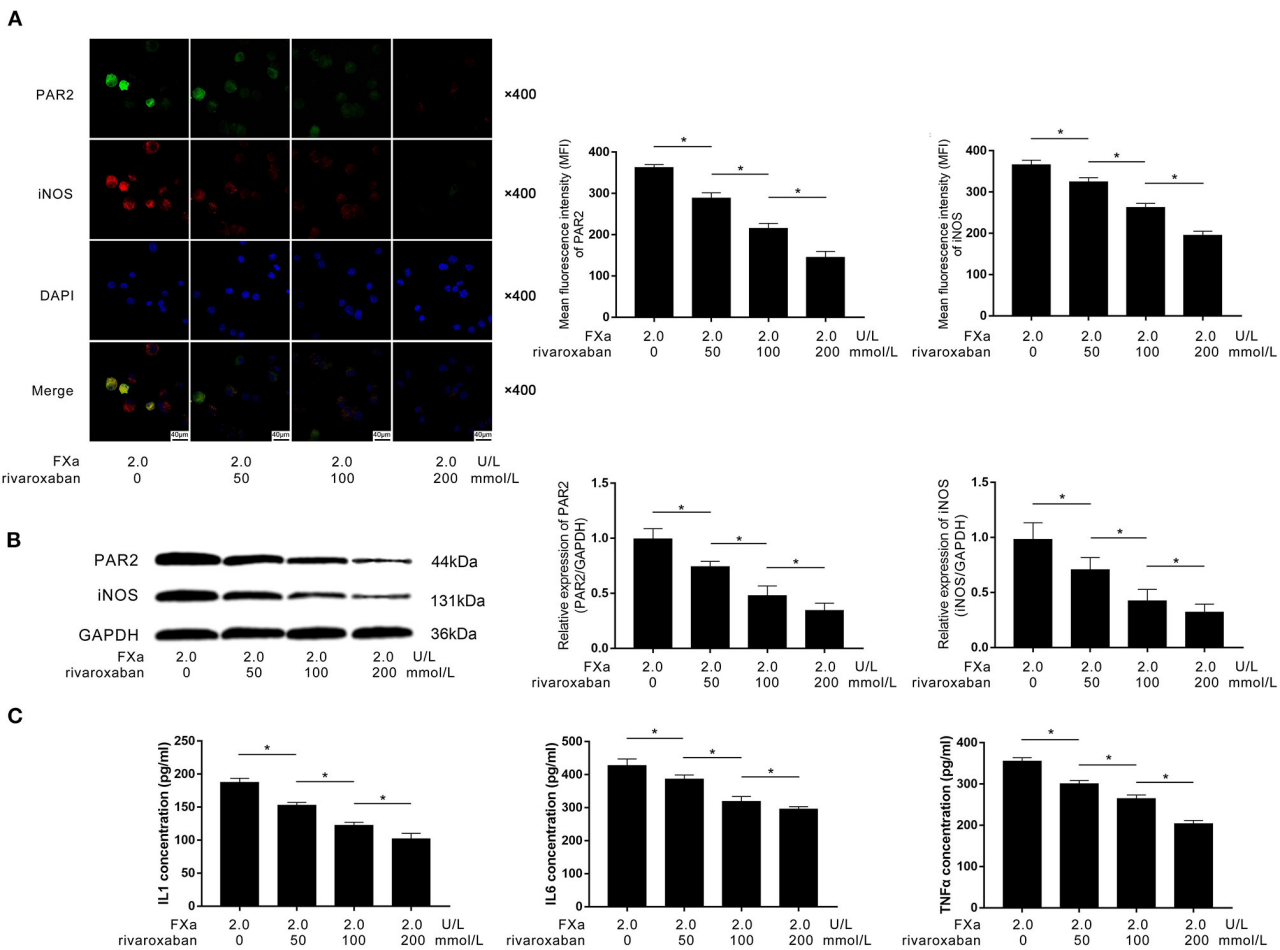
**(A)** Immunofluorescence stains of PAR2, iNOS, ad DAPI and their merged images of macrophages are demonstrated. Columns indicated the mean fluorescence intensity of PAR2 and iNOS of macrophages exposed to FXa at 2.0 U/L and treated with rivaroxaban at concentrations of 0, 50, 100, and 200 mmol/L. **(B)** Immunoblots of PAR2, iNOS, and GAPDH. Columns indicate the relative expression levels of PAR2 and iNOS in macrophages exposed to FXa at 2.0 U/L and treated with rivaroxaban at concentrations of 0, 50, 100, and 200 mmol/L. **(C)** Columns indicate the concentrations of IL1, IL6, and TNFα in the cell culture medium of macrophages exposed to FXa at 2.0 U/L and treated with rivaroxaban at concentrations of 0, 50, 100, and 200 mmol/L (data were presented as mean ± SD, *n* = 6, Student's *t*-test and ANOVA, *P < 0.05).

As the direct inhibitor of FXa, rivaroxaban (final concentrations at 0, 50, 100, and 200 nmol/L) were used to pre-treat the FXa (2 U/L)-exposed macrophages. As demonstrated in Figures 5A,B, rivaroxaban treatment dramatically decreased the expression of PAR2 and M1 phenotypic marker iNOS in FXa-exposed macrophages in a concentration-dependent manner. As demonstrated in Figure 5C, the treatment of rivaroxaban reduced the concentrations of IL1β, IL6, and TNFα in the culture medium of FXa-exposed macrophages in a concentration-dependent manner.

### Rivaroxaban Reduced Dll4 Expression on FXa-Exposed Macrophages by Suppressing PAR2/Akt/HIF1α Pathway

**Figure 6:**
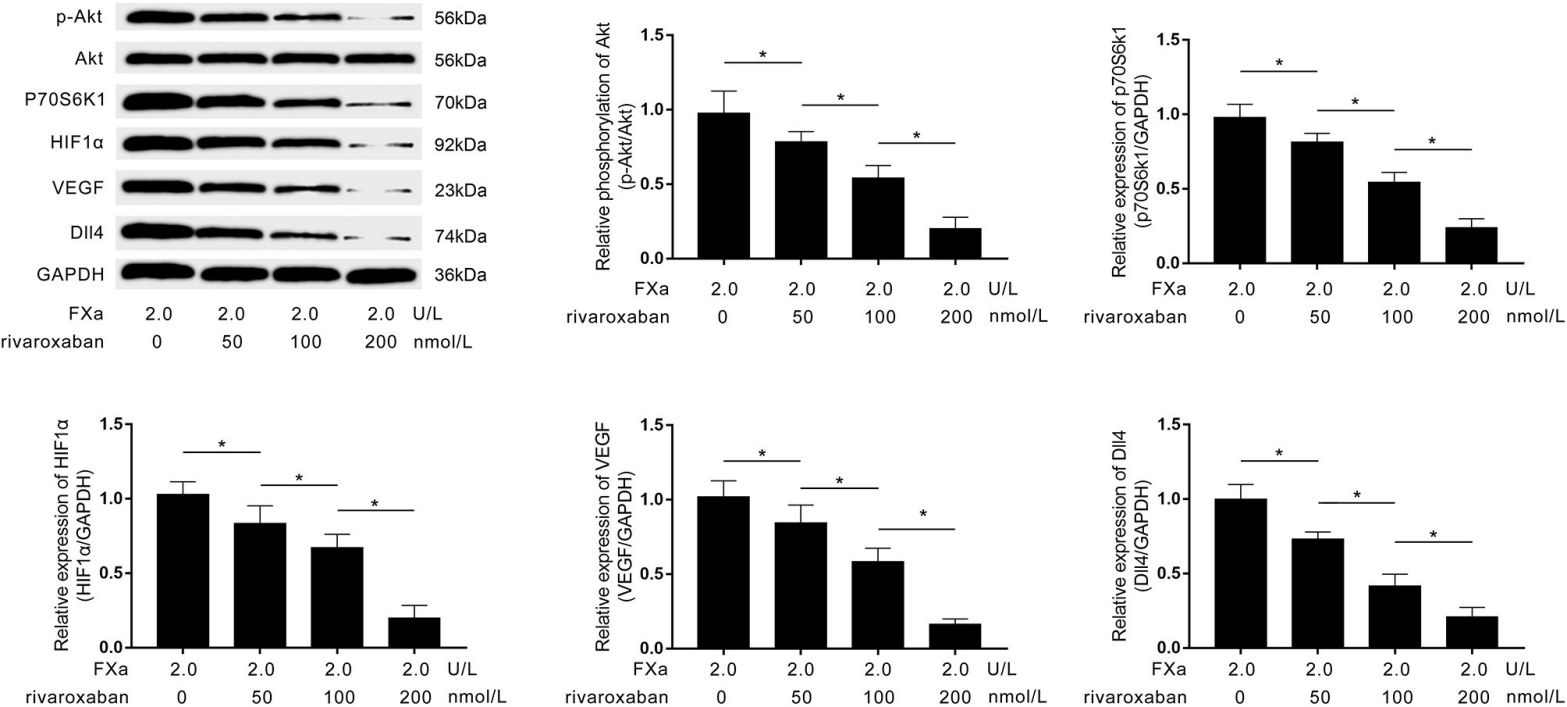
Immunoblots of p-Akt, Akt, p70S6K1, HIF1α, VEGF, Dll4, and GAPDH are demonstrated. Columns indicate the relative expression levels of HIF1α, VEGF, and Dll4, as well as the relative phosphorylation level of Akt in macrophages exposed to FXa at 2.0 U/L and treated with rivaroxaban at concentrations of 0, 50, 100, and 200 mmol/L (data were presented as mean ± SD, *n* = 6, Student's *t*-test and ANOVA, *P < 0.05).

As demonstrated in Figure 6, rivaroxaban treatment decreased the expression levels of p70S6K1, HIF1α, VEGF, Dll4, as well as the phosphorylation levels of Akt in FXa- exposed macrophages in a concentration-dependent manner.

### Macrophage Rivaroxaban Treatment Attenuated VSMCs Phenotypic Conversion in Contact Co-culturing Model

**Figure 7:**
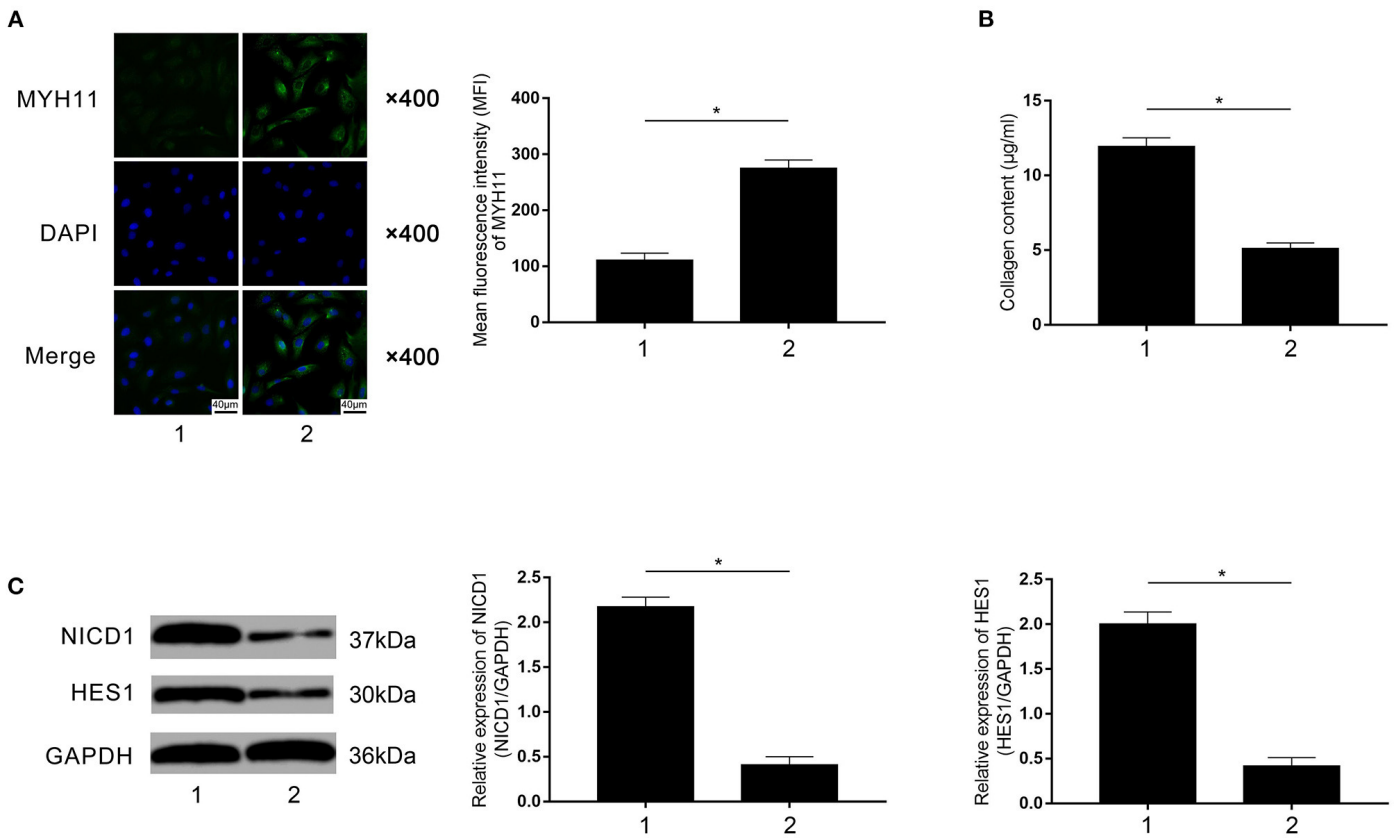
**(A)** Immunofluorescence stains of MYH11, DAPI, and their merged images are demonstrated. Columns indicate the mean fluorescence intensity of MYH11 in VSMCs co-cultured with rivaroxaban-treated or un-treated macrophages exposed to FXa in the contact co-culturing model. **(B)** Columns indicate the measured collagen content of VSMCs co-cultured with rivaroxaban-treated or un-treated macrophages exposed to FXa in the contact co-culturing model. **(C)** Immunoblots of NICD1, HES1, and GAPDH are demonstrated. Columns indicate the relative expression levels of NICD1 and HES1 in VSMCs co-cultured with rivaroxaban-treated or un-treated macrophages exposed to FXa in the contact co-culturing model (data were presented as mean ± SD, *n* = 6, Student's *t*-test and ANOVA, *P < 0.05).

Rivaroxaban-treated FXa-exposed macrophages were employed to co-culture with primary VSMCs in the contact co-culturing system. Compared with rivaroxaban- untreated macrophages (marked as “1”), as demonstrated in Figure 7A, rivaroxaban-treated M1 macrophages significantly increased MYH11 expression in VSMCs (marked as “2”). Figure 7B demonstrated that the collagen content in the medium was decreased in the contact co-culturing model in which the macrophages received rivaroxaban treatment compared with rivaroxaban un-treated macrophages. As demonstrated in Figure 7C, M1 macrophages that received rivaroxaban treatment significantly decreased NICD1 and HES1 expressions levels in VSMCs in the contact co-culturing model. These results indicated that rivaroxaban treatment reversed M1 macrophage-mediated VSMCs contractile-synthetic phenotypic conversion.

## Discussion

It is now accepted that macrophages play important roles in the occurrence and development of AS. M1 macrophages are predominant in developing and unstable plaques, which are characterized by the secretion of pro-inflammatory cytokines and expression of M1 phenotypic marker iNOS (21). Results from our *in vivo* study suggested that M1 macrophages were accumulated in atherosclerotic plaques. The M1 polarization is motivated during various pathological processes including coagulation cascade (22). Results from previous investigations indicated that FXa induced pro-inflammatory responses in macrophages (23). In this study, we found FXa inhibitor rivaroxaban administration significantly reduced macrophage M1 polarization in plaques and eventually inhibited plaque progression. In the *in vitro* study, we found that FXa exposure triggered macrophage M1 polarization and induced the production of pro-inflammatory cytokines including IL1β, IL6, and TNFα, which were inhibited by rivaroxaban administration. These results suggested that FXa played a key role in the progression of AS, which was mediated by M1 macrophages.

The non-hemostatic activities of FXa are largely dependent on the activation of PARs (24). PARs are a family of G-protein-coupled receptors which are activated up on proteolytic cleavage. PAR2 is cleaved and activated by FXa. Activated PAR2 further initiates macrophage M1 polarization (8). In this study, PAR2 was silenced by *PAR2*-siRNA transfection in macrophages. Our results showed that PAR2 silencing inhibited FXa- induced macrophage M1 polarization which was evidenced by downregulation of M1 marker iNOS expression and reduction of pro-inflammatory cytokines. Since M1 macrophages are predominant in AS, we further investigated the possible roles of PAR2-associated signaling pathways.

According to previous studies, PAR2 promoted activation of phosphoinositide 3-kinase (PI3K)/Akt signaling pathway which was related to a wide range of cellular biological processes (25). Phosphorylated Akt activated its downstream target p70S6K1 by phosphorylation (26). It was reported that p70S6K1 knocking-down inhibited HIF1α expression, indicating the role of the Akt/p70S6K1 pathway in non-hypoxic HIF regulation (27). Mediators, such as VEGF, could be released upon the activation of hypoxia HIF1α activation (28, 29). The downstream location of Dll4 of the HIF1α/VEGF signaling was confirmed (10). In the present study, siRNAs were used to silence PAR2, Akt, and HIF1α in FXa-induced M1 macrophages. The results showed that PAR2 silencing inhibited Akt/p70S6K1 activation; Akt silencing suppressed HIF1α/VEGF activation; and HIF1α silencing reduced Dll4 expression. These results indicated PAR2/Akt/HIF1α composed a pathway leading to Dll4 expression up-regulation during the FXa-induced M1 polarization in macrophages.

The prominent role of VSMCs in AS has been well-established. VSMCs are plastic and exhibit different phenotypes in response to various stimuli. During the progression of atherosclerotic plaques, VSMCs conduct contractile-to-synthetic phenotypic conversion. The mechanisms regarding the regulation of the phenotypic conversion of VSMCs is complicated. Previous investigations have suggested that Dll4–Notch signaling was responsible for the phenotypic conversion. In this study, primary VSMCs were co-cultured with FXa-exposed macrophages in both non-contact and contact co-culturing models. FXa-exposed macrophages induced VSMCs contractile-synthetic phenotypic conversion in the contact co-culture model rather than the non-contact co-culture model. Dll4 silencing also made macrophages incompetent in inducing phenotypic conversion of VSMCs in the contact co-culture model. These results indicated that FXa-induced M1 macrophage expressing Dll4 induced phenotypic conversion of VSMCs. This conversion was mediated by the direct contact between macrophages and VSMCs through Dll4–Notch signaling.

As the FXa direct inhibitor, rivaroxaban is an approved anticoagulant widely used in the prevention and treatment of thromboembolism. Other than the anticoagulant activity, rivaroxaban also showed regulatory impacts on the PAR2 signaling pathway, which is activated by FXa. The previous investigation proposed that PAR2 deficiency attenuated atherosclerotic plaque formation (30). In our *in vivo* investigation, we found that rivaroxaban administration significantly inhibited atherosclerotic plaque progression. In the *in vitro* part, FXa-exposed macrophages were treated with rivaroxaban. The results showed that rivaroxaban effectively attenuated FXa-induced macrophage polarization toward M1. Moreover, by deactivating PAR2/Akt/HIF1α signaling pathway, rivaroxaban treatment reduced the expression of Notch ligand Dll4 in FXa-exposed macrophages. As a result, rivaroxaban treatment inhibited FXa-exposed macrophage-mediated contractile-synthetic phenotypic conversion of VSMCs in the contact co-culture model by suppressing Notch signaling. In our *in vivo* study, rivaroxaban treatment blocked both macrophage M1 polarization and contractile-synthetic phenotypic conversion of VSMCs in atherosclerotic plaques. These results suggested that rivaroxaban was capable of suppressing the phenotypic conversion of VSMCs by reducing macrophage Dll4 expression during the process of FXa-induced M1 polarization.

## Conclusion

In summary, this present study suggested that FXa-induced macrophage M1 polarization was mediated by PAR2 signaling, which endowed macrophages with the characteristic of expressing Notch ligand Dll4. By direct contact, the above M1 macrophages induced contractile-synthetic phenotypic conversion of VSMCs by activating Notch signaling which contributed to the progression of atherosclerotic plaques. By direct inhibition of FXa, rivaroxaban treatment reduced Dll4 expression in macrophages *via* suppressing PAR2/Akt/HIF1α signaling. As a result, rivaroxaban-treated M1 macrophages were incompetent in inducing contractile-synthetic phenotypic conversion of VSMCs after direct cell-to-cell contact. Results from this study not only enriched our current understanding of the mechanisms of AS but also provided new clues and evidence to the potential application of rivaroxaban in the treatment of AS. However, further in-depth studies are still needed to confirm the clinical relevance of our findings.

### Limitation

The current study has several limitations. First, data from the *in vivo* study was not sufficient to demonstrate the role of PAR2 in AS. Employment of PAR2 knock-out or knock-in mice would make the study more persuasive. Second, when investigating the phenotypic conversion of VSMCs *in vivo*, it would be better to use lineage tracing reporter mice in which all the VSMCs could be labeled no matter what kind of phenotype of VSMCs are present.

## Data Availability Statement

The raw data supporting the conclusions of this article will be made available by the authors, without undue reservation.

## Ethics Statement

The animal study was reviewed and approved by Institutional Ethics Committee for Animal Use and Experiment of Shaanxi Provincial People's Hospital.

## Author Contributions

YM was involved in the conceptualization, experiments, formal analysis, writing the original draft, analysis, and writing. YZ was involved in the experiments, writing the original drafts, analysis, and writing. CQ took care of data curation and analysis. CH and TH were involved in the experiments. SS was involved with conceptualization, experiments, data curation, and data acquisition. ZL was involved in the conceptualization, experiments, writing the original draft, analysis, and writing. All authors contributed to the article and approved the submitted version.

## Funding

This study was supported by the Health Research Project of Shaanxi Province (Number: 2018E11), the National Natural Science Foundation of China (Number: 82070858), and SPPH Scientific Research Support Project (2021BJ-02).

## Conflict of Interest

The authors declare that the research was conducted in the absence of any commercial or financial relationships that could be construed as a potential conflict of interest.

## Publisher's Note

All claims expressed in this article are solely those of the authors and do not necessarily represent those of their affiliated organizations, or those of the publisher, the editors and the reviewers. Any product that may be evaluated in this article, or claim that may be made by its manufacturer, is not guaranteed or endorsed by the publisher.
